# Kleptoplasty: Getting away with stolen chloroplasts

**DOI:** 10.1371/journal.pbio.3001857

**Published:** 2022-11-08

**Authors:** Sónia Cruz, Paulo Cartaxana

**Affiliations:** Laboratory for Innovation and Sustainability of Marine Biological Resources (ECOMARE), Centre for Environmental and Marine Studies (CESAM), Department of Biology, University of Aveiro, Aveiro, Portugal

## Abstract

Kleptoplasty, the process by which a host organism sequesters and retains algal chloroplasts, is relatively common in protists. The origin of the plastid varies, as do the length of time it is retained in the host and the functionality of the association. In metazoa, the capacity for long-term (several weeks to months) maintenance of photosynthetically active chloroplasts is a unique characteristic of a handful of sacoglossan sea slugs. This capability has earned these slugs the epithets “crawling leaves” and “solar-powered sea slugs.” This Unsolved Mystery explores the basis of chloroplast maintenance and function and attempts to clarify contradictory results in the published literature. We address some of the mysteries of this remarkable association. Why are functional chloroplasts retained? And how is the function of stolen chloroplasts maintained without the support of the algal nucleus?

## Abstract 2

Why do some animals steal chloroplasts from their food? And how is the function of stolen chloroplasts (known as kleptoplasts) maintained? This Unsolved Mystery explores the basis of kleptoplast maintenance and function and attempts to clarify contradictory results about kleptoplasty in the published literature.

## Introduction

Kleptoplasty is the capacity of a non-photosynthetic organism to acquire and maintain algal chloroplasts [1]. The process involves digestion of the algal cellular components by the host organism, including the nucleus, while the chloroplasts—thereafter termed kleptoplasts—are maintained structurally intact and, in many cases, photosynthetically competent [1,2]. This extraordinary act of thievery is sometimes referred to as endosymbiosis, or one organism living within another, although this is not accurate in this case as the chloroplast does not constitute an organism [3]. Nevertheless, kleptoplast-bearing organisms have emerged as potential model species in the study of the early stages of the endosymbiosis that led to chloroplast establishment [4].

The chloroplast derives from primary endosymbiosis involving the uptake of a cyanobacterium by a non-photosynthetic eukaryote more than 1.5 billion years ago [5]. This has allowed host organisms to convert light into energy and has deeply impacted the evolution of life [6]. Secondary and tertiary plastid acquisition events, among several eukaryote lineages, gave rise to the diversity of algae found in aquatic ecosystems [7,8]. The transformation into an obligate organelle entailed a considerable reduction in the genome size of the endosymbiont, partly due to gene transfer into the nuclear genome of the host [9]. Hence, many of the proteins required for the functioning and maintenance of the photosynthetic apparatus in today’s photosynthetic algae are nucleus encoded [8–10]. One of the unsolved mysteries of kleptoplasty, particularly in animal cells, is how the host is able to maintain algal chloroplasts in a photosynthetically competent state for long periods, after stripping them of their nuclear entourage.

Kleptoplasty is not without risks. Reactive oxygen species (ROS) are generated in the chloroplasts as a result of the photosynthetic transport of electrons and the transfer of energy, particularly under strong light [11]. ROS act mainly by inhibiting the de novo synthesis of proteins, which compromises the repair of photosystem II (PSII) [12] (Box 1). In host organisms harboring kleptoplasts, ROS formation can compromise the functionality of the stolen organelle, but damages may also extend to the rest of the host cell [4]. Considering that chloroplast acquisition is a risky mission for the host, it is reasonable to assume that the gains of a partially photosynthetic lifestyle must be considerable. However, the actual benefits of harboring stolen chloroplasts are still somewhat controversial.

**Box 1. Glossary**

**Monophagous**

Feeding on one kind of food, such as on a certain type of plant or algae.

**Paraheliotropism**

Leaf movement in response to incoming light, usually minimizing excess light absorption.

**Peridinin**

Carotenoid pigment found in the light-harvesting antenna of dinoflagellates.

**Photic zone**

The upper layer of a body of water in which enough sunlight can penetrate to permit photosynthesis.

**Photosystem I**

Membrane protein complex (plastocyanin–ferredoxin oxidoreductase) that uses light energy to catalyze the transfer of electrons across the thylakoid membrane, as part of the light-dependent reactions of oxygenic photosynthesis.

**Photosystem II**

Membrane protein complex (water–plastoquinone oxidoreductase) that uses light energy to split H_2_O into O_2_, protons, and electrons, as part of the light-dependent reactions of oxygenic photosynthesis.

**Zooxanthellae**

Informal term for single-celled dinoflagellates that are able to live in symbiosis with different marine invertebrates (e.g., corals, jellyfish, and nudibranchs).

In this Unsolved Mystery, we identify the taxa in which kleptoplasty has been reported and review the most relevant literature on a peculiar group of metazoans, the sacoglossan sea slugs. We aim to address some of the unsolved mysteries of this remarkable association between an animal and an algal-derived organelle.

### Which organisms steal chloroplasts from algae?

Kleptoplasty occurred on multiple occasions in distantly related protist lineages, such as dinoflagellates, ciliates, and foraminifera [7,13–15]. In these protist lineages, the origin of the stolen algal chloroplasts is diverse, including diatoms, prasinophytes, haptophytes, and cryptophytes [7]. In dinoflagellates, chloroplast origin is particularly complex and highly chimeric [16]. Several lineages of dinoflagellates have been identified to maintain long-term photosynthetically active kleptoplasts. The Antarctic Ross Sea dinoflagellate, which is able to partition functions between ancestral peridinin plastids and chloroplasts stolen from its haptophyte prey [17], is able to maintain active kleptoplasts for as long as 30 months [18]. Other dinoflagellates have cryptophyte-derived or diatom-derived kleptoplasts. In one of the most peculiar cases, *Dinophysis* obtains its kleptoplasts by feeding on the ciliate *Mesodinium rubrum*, which in turn acquires the plastids from cryptophytes [19]. Several authors have classified kleptoplasty in some dinoflagellates as an emerging endosymbiotic event or a transitional phase towards permanent chloroplasts [19,20].

Chloroplast-bearing foraminifera are usually shallow-water species, inhabiting the photic zone, that acquire their plastids mostly from diatoms [15,21]. In some foraminifera species, the kleptoplasts break down within hours or days; while in other species, they remain functional for weeks to months [21–23]. Inorganic carbon labeling experiments have shown light-dependent incorporation in some foraminifera, an indication that kleptoplast photosynthesis constitutes an additional carbon source for these organisms [24,25]. However, kleptoplasty was also observed in deep-sea species (approximately 600 m), where light levels are too low to fuel photosynthesis [23,26]. In the latter case, the chloroplasts were intact and functional for up to 1 year [23]. Given that foraminifera are unable to acquire and assimilate inorganic sources of nitrogen, it was suggested that the kleptoplasts were used to fulfil the nitrogen requirements of the host [23].

Among metazoans, kleptoplasty is the privilege of a few. Until recently, the animal chloroplast-stealing gang was restricted to sacoglossan sea slugs, but it has been extended to include rhabdocoel flatworms [27]. In the latter organisms, kleptoplasts originated from different diatoms, and photosynthetic activity was maintained for only a few days [27]. The capacity for long-term (several weeks to months) maintenance of photosynthetically active chloroplasts, potentially for the entire lifespan of the host, is a unique characteristic of a handful of Sacoglossa, mostly within the genus *Elysia* [28,29]. Some of these sea slugs are monophagous, sequestering chloroplasts from a specific algal species. The sea slugs with the longest retention times of functional chloroplasts, *Elysia chlorotica* and *Elysia timida*, feed on and retain chloroplasts exclusively from the chromophytic alga *Vaucheria litorea* and the green alga *Acetabularia acetabulum*, respectively [29,30]. The sacoglossans *Elysia viridis*, *Elysia crispata*, and *Plakobranchus ocellatus* are less picky with their food, obtaining chloroplasts from a variety of different algae, mostly siphonous green seaweeds [31–33]. In the next sections of this Unsolved Mystery, we focus on these best-studied, successful long-term animal–chloroplast partnerships.

### Why do some sacoglossan sea slugs retain stolen functional chloroplasts?

**Fig 1:**
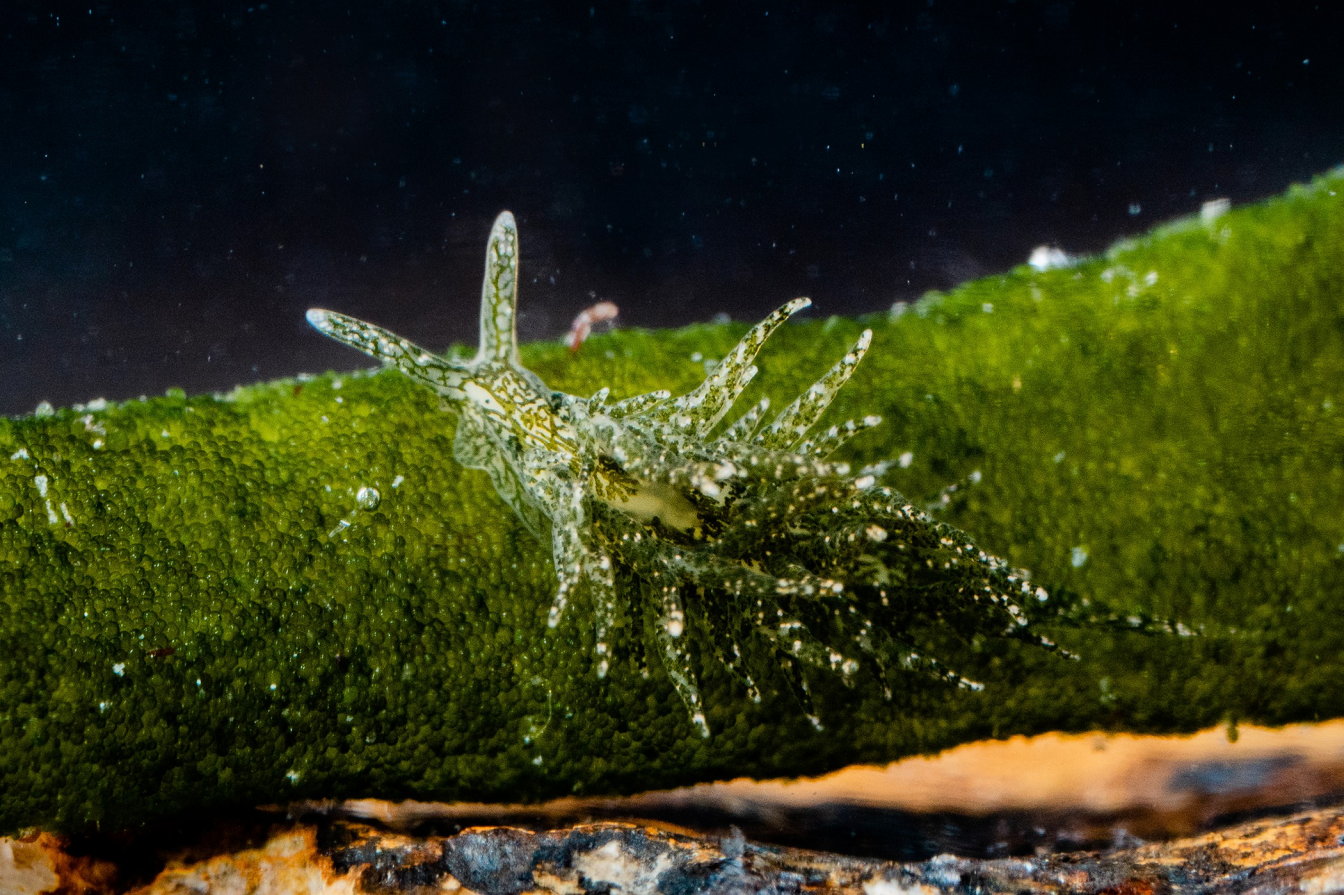
Crypsis in sacoglossan sea slugs. The sea slug *Placida dendritica* retains short-term, nonfunctional kleptoplasts that allow the sea slug to blend with the environment and avoid predation.

In kleptoplast-bearing sea slugs, the macroalgal chloroplasts are phagocytized by cells of the digestive tubules, which ramify throughout most of the sea slug’s body [34]. Hence, one of the most noticeable characteristics of these sea slugs is their green coloration, which allows them to blend with the environment dominated by macroalgae and pass unnoticed to predators such as fish and crabs [35,36]. Avoiding predation might have been an important evolutionary drive in the acquisition of chloroplasts by some sacoglossans. However, crypsis is also an ability of sea slugs that acquire nonfunctional chloroplasts, such as *Placida dendritica* (Fig 1). These chloroplasts are maintained in the animal cells for just a few days and are not photosynthetically competent [28]. Therefore, crypsis alone cannot explain the evolution of long-term retention of functional chloroplasts in Sacoglossa.

Earlier studies using inorganic labeled^14^C indicate that photosynthesis-derived metabolites are translocated from the kleptoplasts in the digestive tubules into kleptoplast-free sea slug tissues [37,38]. NanoSIMS (Nanoscale Secondary Ion Mass Spectrometry), which allows high-resolution measurements of elemental and isotopic ratios, was recently used to image carbon translocated from kleptoplasts to the reproductive organs of 2 different sacoglossans [39,40]. In the following sections, we will evaluate some of the advantages related to the translocation of photosynthates from kleptoplasts to sea slug cells, which may help explain this unique evolutionary trait.

### Longer survival in periods of food shortage

The great majority of studies addressing the role of kleptoplast photosynthesis in sacoglossan sea slugs typically compare weight and survival rates of starved specimens (unable to feed) under dark (unable to acquire photosynthates from the kleptoplasts) and light conditions [41–46]. Evidence of kleptoplast photosynthesis minimizing weight loss and increasing survival in periods of food scarcity in sea slugs such as *E*. *viridis*, *E*. *timida*, *E*. *chlorotica*, and *P*. *ocellatus* are overwhelming [41–45]. On the contrary, 1 single study reported comparable weight loss in *P*. *ocellatus* starved under dark and light conditions and in the presence of a photosynthesis inhibitor [46]. However, that study’s conclusion that kleptoplast photosynthesis has no impact on the survival of sacoglossan sea slugs during periods of food shortage was likely overstated, as the authors based it on an extremely low sample size (2 slugs per condition) and failed to show a fed control treatment in the light. Although metabolites produced by kleptoplast photosynthesis are continuously made available to the sea slugs by rapid translocation into kleptoplast-free tissues [37–40], it is also possible that under prolonged starvation sea slugs obtain nutritive benefits by targeting kleptoplasts for degradation and using their starch reserves [46,47].

It is important to note that extended periods of complete darkness are ecologically unrealistic [48]. On the other hand, long starvation treatments have an impact on both heterotrophic and autotrophic nutrition because in the absence of the macroalgal food source, the older kleptoplasts are not replaced and the photosynthetic activity declines [49]. Overcoming these limitations, Baumgartner and colleagues [48] observed increased growth efficiency in *E*. *viridis* fed on *C*. *fragile* under regular light compared to quasi-dark, which correlated to increased photosynthesis. By contrast, *E*. *viridis* feeding on *Cladophora rupestis* did not display an increase in growth efficiency under light conditions due to the highly limited functionality of the algal chloroplasts. Using stable nitrogen isotopic composition of amino acids, the nutritional role of photosynthesis in wild *P*. *ocellatus* feeding on multiple macroalgae was found to be negligible [50]. Nevertheless, the authors concluded that kleptoplast photosynthates formed a significant nutritional source for animals in periods of food shortage. Based on the mass balance of stable carbon isotopes at the natural abundance level, total carbon derived from kleptoplast photosynthesis was estimated to range between 16% and 60% in several Sacoglossa [51].

### Higher reproductive output

**Fig 2:**
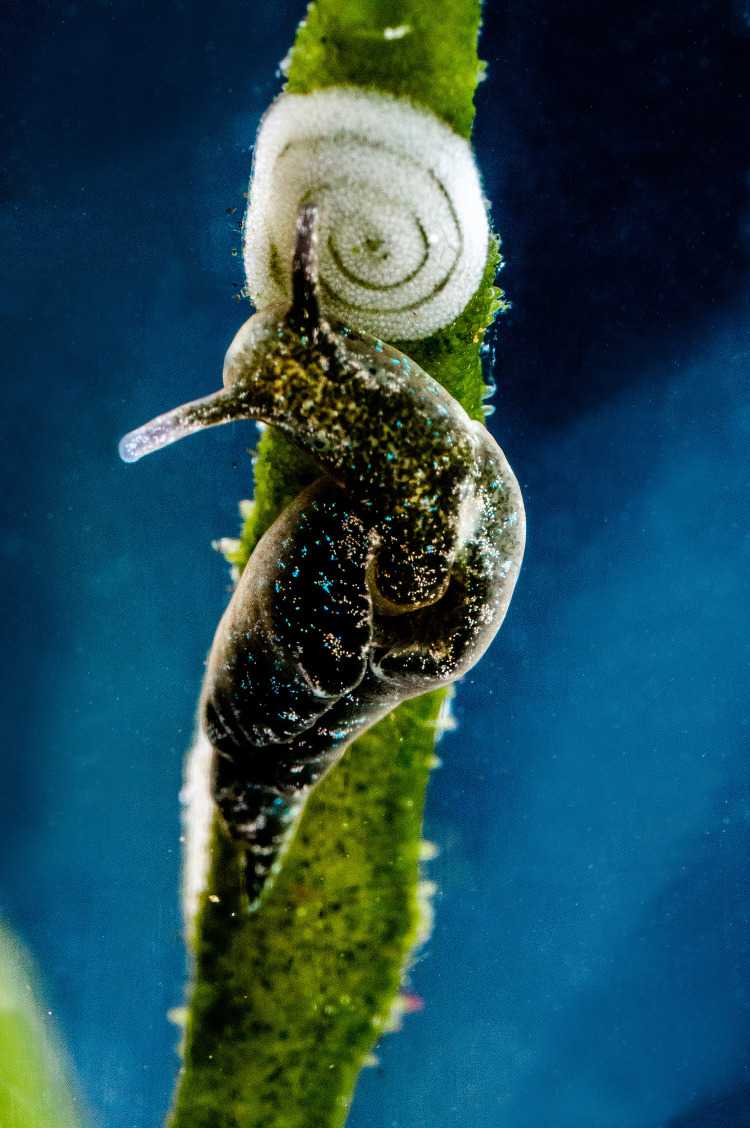
Photosynthesis and reproductive output. *Elysia viridis* spawning an egg mass on the macroalga *Codium tomentosum*. Photosynthesis by long-term functional kleptoplasts can support the reproductive output of the host slug.

It was recently hypothesized that kleptoplast photosynthesis could support the reproductive output of sacoglossan sea slugs [39,40]. It is reasonable to consider that better-nourished individuals, including sea slugs able to obtain metabolites from kleptoplast photosynthesis, have more resources to allocate to reproduction and consequently display higher fecundity (Fig 2).

The sea slug *E*. *atroviridis* was shown to spawn a higher number of eggs when fed under regular light than under quasi-dark conditions [52]. Using NanoSIMS, it was possible to image light-dependent incorporation of ^13^C and ^15^N in the albumen gland and gonadal follicles of the sea slugs *E*. *viridis* and *E*. *timida* [39,40]. The authors demonstrated that inorganic carbon and nitrogen assimilated by the kleptoplasts in the digestive tubules were subsequently translocated to the reproductive organs of the sea slugs. Furthermore, it was shown that long-chain polyunsaturated fatty acids with reported roles in reproduction were produced in the sea slug cells using labeled precursors translocated from the kleptoplasts [39,40]. In addition, the number of eggs spawned by *E*. *timida* during a 4-week period was significantly higher in sea slugs exposed to regular light than in animals reared under quasi-dark [40]. Under limited kleptoplast photosynthesis due to reduced light levels, the sea slugs clearly changed their reproductive energy investment by decreasing the number of spawned eggs [40].

### Increased mucus production

Mucus secretion is paramount for mollusks, playing different roles in processes such as locomotion, feeding, reproduction, or protection, including reducing exposure to predation or physical stress [53]. A relevant role of kleptoplast photosynthesis in providing substrates for mucus production by sacoglossan sea slugs has been hypothesized [54–56]. Using ^14^C-labeling experiments, Trench and colleagues [37] showed carbon incorporation in the mucus-secreting pedal gland of *E*. *crispata* and *E*. *diomedea*. Estimates of carbon fixed by the kleptoplasts that end up in the secreted mucus ranged between 5% and 30% in the sea slugs *E*. *crispata*, *E*. *diomedea*, and *P*. *ocellatus*, with most of the labeled carbon incorporated into galactose and glucose [54,55]. Limiting photosynthesis by rearing sea slugs under reduced light led to lower mucus production and lower carbohydrate concentrations in the secreted mucus than under regular light [56].

In symbiotic reef corals, the release of mucus has often been associated with phototrophic nutrition [57–59]. Increased photosynthetic activity of the zooxanthellae with higher light availability lead to greater translocation to the coral host of high-energy compounds, which are channeled into coral respiration and mucus production [58,59]. Further studies are required to determine the relevance of kleptoplast-derived metabolites in mucus production by sacoglossan sea slugs.

### How do photosynthetic sea slugs avoid or repair oxidative damage in the stolen chloroplasts?

**Fig 3:**
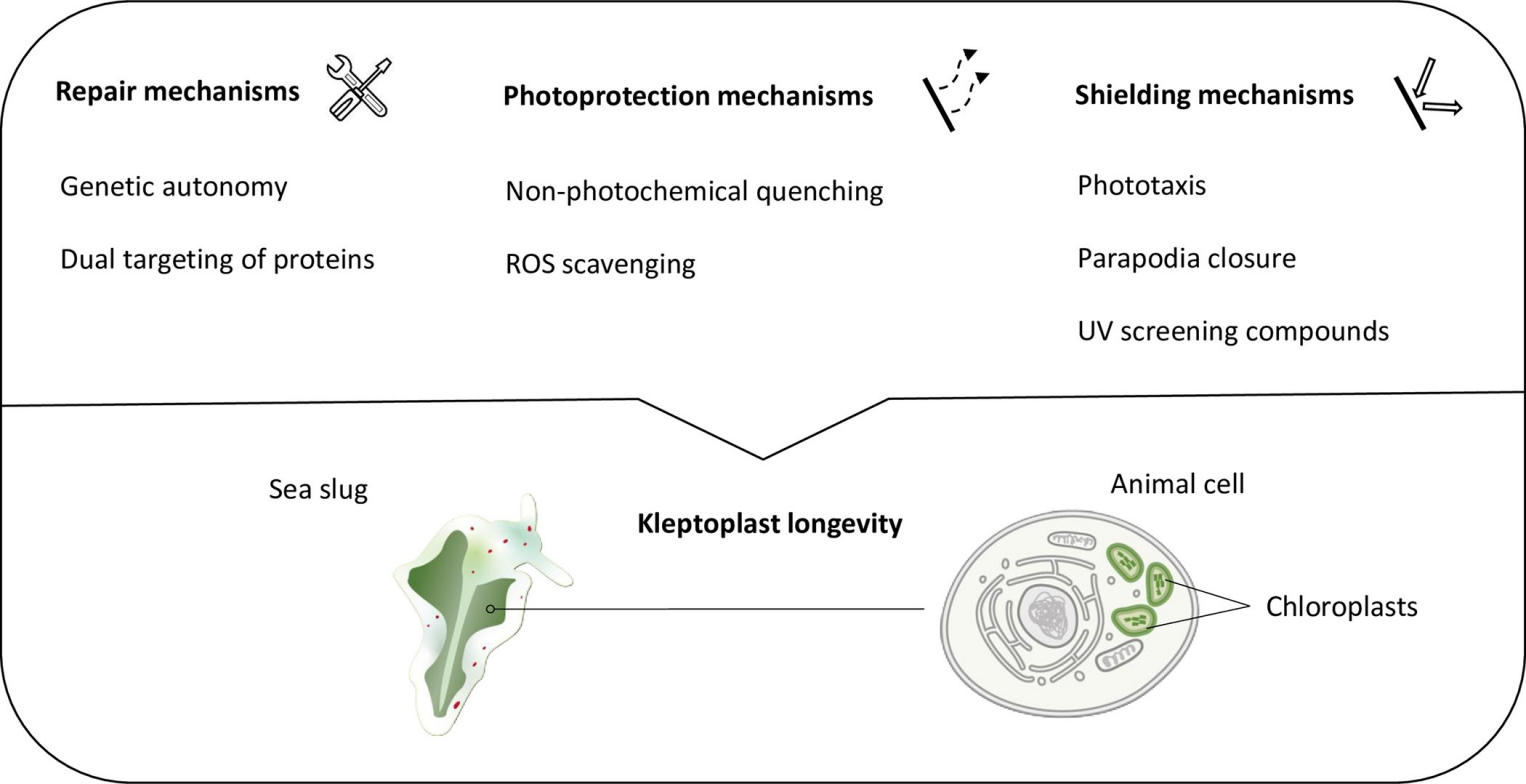
Repair and avoidance mechanisms involved in kleptoplast longevity. Schematic representation of repair, photoprotection, and shielding mechanisms putatively involved in the long-term maintenance of photosynthetic active chloroplasts in sacoglossan sea slugs.

The most controversial hypothesis put forward to answer this question involves horizontal gene transfer (HGT) of algal nuclear genes encoding essential chloroplast proteins to the host nuclear genome [60–62]. Earlier evidence of HGT from the alga *V*. *litorea* to the nuclear DNA of the sea slug *E*. *chlorotica* was contradicted by reports of the absence of algal-derived genes in the germline of the sea slug [63]. In accordance, genomic and transcriptomic studies on the sea slugs *E*. *timida* and *P*. *ocellatus* found no evidence of HGT [64,65]. Hence, sacoglossan sea slugs do not seem to introduce foreign algal genes to support their photosynthetic lifestyle [63–65]. Alternative hypotheses to HGT need to be considered to explain the long-term maintenance of functional kleptoplasts in sacoglossans (Fig 3).

### Chloroplast robustness and genetic autonomy

Chloroplasts isolated from the alga *Codium fragile*, one of the chloroplast sources of *E*. *viridis*, were shown to be more stable than spinach chloroplasts, fixing CO_2_ for several days after isolation [66]. The robustness of siphonous algae chloroplasts could be an important factor in the ability to integrate and function in sea slug cells [66]. Similar results were later reported for *V*. *litorea* chloroplasts, which were also largely unaffected by osmotic fluctuations [67]. The reasons underlying the robustness of these chloroplasts remain unclear, but high capacity for repair of the light-damaged PSII has been considered crucial for kleptoplast longevity in sacoglossan sea slugs [68,69].

In the algae *V*. *litorea* and *A*. *acetabulum*, the protease FtsH, essential for PSII repair, is encoded in the chloroplast genome [60,69]. In land plants, the *ftsH* gene has been transferred to the nuclear genome and the protein needs to be imported from the cytosol [69]. The FtsH protease is responsible for the removal of the photodamaged core protein D1 of PSII, before the insertion of a newly synthesized D1 that is encoded by the plastome gene *psbA*. It was previously shown that chloroplasts sequestered from *V*. *litorea* by the sea slug *E*. *chlorotica* continue to actively transcribe and translate the plastid-encoded *psbA* [70]. The D1 repair cycle reduces the accumulation of damaging ROS that would rapidly limit photosynthesis [11,71,72]. Evidence for the presence of transcripts of *ftsH*, *psbA*, and *tufA* genes in *E*. *timida* were reported in sea slugs that had been starved for 1 month [69]. The translation of *ftsH* transcript would be impaired in the absence of the crucial elongation factor Tu encoded by *tufA* [69]. Assessing the transcripts of 7 plastome genes in isolated *V*. *litorea* chloroplasts, Havurinne and colleagues [73] observed that levels of *ftsH* and *tufA* decreased slower than the transcripts of the other tested genes. Furthermore, photoinhibition in *V*. *litorea* was similar in the absence and presence of a cytosolic translation inhibitor, suggesting that the PSII repair cycle was not dependent on active translation of nuclear-encoded proteins [73]. These chloroplasts seem to possess the genetic autonomy for maintaining a PSII repair cycle.

### Physiological photoprotective mechanisms

Photosynthetic organisms display a variety of physiological photoprotective mechanisms that can protect the photosynthetic apparatus after light is absorbed by the light-harvesting systems, but before actual damage is inflicted by ROS. The photoprotective mechanism prevalent in high light levels is the conversion and dissipation of the excess excitation energy as heat, typically assessed as the energy-dependent component of non-photochemical quenching (NPQ), qE [74]. This fast reversible NPQ component requires the irradiance-dependent establishment of a transthylakoidal proton gradient and the activation of a xanthophyll cycle (XC) [75,76]. A fast reversible NPQ component and functional XCs were reported in kleptoplasts of *E*. *timida* and *E*. *chlorotica* [77–79]. Furthermore, both *E*. *timida* and *E*. *chlorotica* were shown to induce NPQ faster and to higher levels than their algae prey [77,79]. In the case of *E*. *timida*, the enhanced NPQ of the sea slugs was related to a stronger acidification of the thylakoid lumen of the kleptoplasts [79]. However, energy dissipation via the XC decreased as *E*. *timida* kleptoplasts aged along with animal starvation, indicating a progressive loss of photoprotective capacity [78].

In the dark, the first component of the photosynthetic electron transport chain, plastoquinone, was found to be more oxidized in the chloroplasts sequestered by *E*. *timida* than in those of its algal prey, *A*. *acetabulum* [79]. Upon transition to light, this allows PSII to transfer extra electrons to plastoquinone, avoiding the buildup of harmful ROS. Furthermore, kleptoplasts of *E*. *timida*, as well as chloroplasts of *A*. *acetabulum*, may use alternative electron sinks (e.g., flavodiiron proteins) to protect the photosystem I (PSI) from photoinhibition [79].

Once ROS are formed, scavenging mechanisms can be triggered to reduce oxidative stress. The sister species *E*. *cornigera* and *E*. *timida* obtain active chloroplasts from the alga *A*. *acetabulum*, but only the latter slug is able to maintain long-term photosynthetically active plastids [80]. Comparing the response of these species to starvation and light stress, de Vries and colleagues [81] observed that *E*. *cornigera* individuals died while accumulating high levels of ROS in the kleptoplast-bearing digestive tubules. Low levels of ROS and *E*. *timida* longer survival could be related to more effective ROS scavenging mechanisms. Isolated thylakoids of *V*. *litorea* were found to be more resilient to photoinhibition of PSII than spinach thylakoids, possible due to lower ^1^O_2_ production and higher concentrations of ROS detoxification compounds (e.g., α-tocopherol and carotenoids) [73]. The maintenance of photoprotective mechanisms (e.g., non-photochemical processes and ROS scavenging) following chloroplast incorporation into sea slug cells could have a significant role in the longevity of kleptoplast photosynthetic activity by reducing light-induced oxidative stress [77,81].

### Chloroplast shielding mechanisms

The 2 previous sections have analyzed the repair and physiological photoprotective mechanisms of kleptoplasts after light is captured by the organelle’s light-harvesting systems. However, an array of mechanisms that avoid light reaching the kleptoplasts can help explain the long-term maintenance of functional plastids in sacoglossan sea slugs. To discriminate from the previously discussed photoprotective processes, we will refer to them as chloroplast shielding mechanisms.

Contrary to sessile photosynthetic life forms, sea slugs are motile and can actively search for optimum light levels. Positive phototaxis has been shown for several sea slug species hosting functional kleptoplasts [82–84]. However, avoidance of high light levels and a preferential selection of irradiance levels coincident with the optimum for photosynthetic activity were also reported [82–84]. Furthermore, all sacoglossan sea slugs that harbor long-term functional chloroplasts have parapodia, wing-like extensions that, when closed, provide shielding from high irradiance [28]. Avoidance of high light levels through parapodia closure has been observed in *E*. *timida*, *E*. *viridis*, and *P*. *ocellatus* sea slugs [78,84,85]. Parapodia closure could be an efficient strategy to reduce absorption under high light availability, functionally equivalent to leaf movement or paraheliotropism in plants [86].

Production of structurally complex compounds that can protect the chloroplasts from the highly photoinhibitory ultraviolet (UV) radiation has been reported in sacoglossan sea slugs [87,88]. In fact, UV-protective long-chain polypropionates were found exclusively in sea slugs with long-term chloroplast retention [87]. In the sea slug *P*. *ocellatus*, incorporation of ^14^C into UV-screening polypropionates was observed, indicating a role of kleptoplast photosynthesis in the biosynthesis of these photoprotective secondary metabolites [88]. UV radiation screening was observed in the tissues of the sea slug *E*. *timida*, preventing it from reaching the kleptoplasts [89]. Hence, efficient UV-screening compounds may greatly improve kleptoplast longevity in sacoglossan sea slugs by reducing damages to the photosynthetic apparatus.

## Conclusion

Studying kleptoplastic sacoglossan sea slugs in isolation from sequestered chloroplasts is extremely complex, as the relation is described as obligatory. The sea slugs will not complete the initial steps of development in the absence of their algal prey and the acquisition of functional chloroplasts [29,43]. Recent transcriptomic data on the early stages of *E*. *chlorotica* development indicated the engagement of communication and complementarity of gene functions between the host and the kleptoplasts [90]. The authors showed that chloroplast sequestration influenced host gene expression in a similar way to the establishment of symbiosis in corals. This strongly suggests that the kleptoplasts are not slowly digestible “snacks” as suggested by some, but rather energy powerhouses that support and are integrated into animal development [90].

It is important to highlight that compatibility between host and algae, enabling kleptoplasty and determining the longevity of kleptoplasts, can be determined by a chain of factors, ranging from feeding preference to intracellular metabolic and immunological selection. Several promising lines of research that were not addressed in this Unsolved Mystery can deepen our understanding of long-term kleptoplasty in sacoglossan sea slugs. For example, the redirecting of animal nuclear-encoded proteins to the kleptoplasts. In fact, there are several mitochondrial proteins (electron transport system, ATP synthase complex) and cytosolic proteins (gluconeogenesis and pentose phosphate pathways) that could replace similar proteins in the kleptoplasts [91,92]. An important hypothesis that remains to be addressed is whether the host provisioning of different compounds enhances kleptoplast longevity.

Regarding the host’s benefits, the involvement of kleptoplasts in the sea slug’s capacity to acquire nitrogen, as has been suggested for deep-sea foraminifera [23], should be further investigated. Light-dependent assimilation of nitrogen was observed from incubations of *E*. *viridis* and *E*. *timida* with ^15^N-labeled nitrogen substrates [39,40,93]. Furthermore, nitrogen assimilation decreased significantly when sea slugs were incubated with specific inhibitors of glutamine (GS) and glutamate synthetases (GOGAT), showing that at least part was occurring through the kleptoplast GS-GOGAT activity [93]. Comparative omics analysis across different sacoglossan sea slug species and different stages of chloroplast integration are necessary to continue the process of unraveling the maintenance of long-term functional kleptoplasts and establish the full benefits of this extraordinary association [90,94].

## Abbreviations

ATP: adenosine triphosphate
GOGAT: glutamate synthetase
GS: glutamine synthetase
HGT: horizontal gene transfer
NanoSIMS: Nanoscale Secondary Ion Mass Spectrometry
NPQ: non-photochemical quenching
PSI: photosystem I
PSII: photosystem II
ROS: reactive oxygen species
UV: ultraviolet
XC: xanthophyll cycle
